# Excessive workload as a risk factor for patient and medical staff safety: a multicenter cross-sectional study in Central Europe

**DOI:** 10.3389/fpubh.2026.1851838

**Published:** 2026-05-28

**Authors:** Katarzyna Tomaszewska, Krystyna Kowalczuk, Helena Kadučáková, Anna Krátká, Erika Durejova, Krzysztof Kalita, Bożena Majchrowicz

**Affiliations:** 1 Faculty of Health Care, The Bronisław Markiewicz State University of Technology and Economics in Jarosław, Jarosław, Poland; 2 Department of Integrated Medical Care, Medical University of Białystok, Białystok, Poland; 3 Department of Nursing, Faculty of Health Sciences, Catholic University in Ružomberok, Ružomberok, Slovakia; 4 Department of Health Care Sciences, Faculty of Humanities, Tomas Bata University in Zlin, Zlín, Czechia; 5 Central Military Hospital in Ružomberok, Ružomberok, Slovakia; 6 Bureau of Statistical Research and Analysis, Rzeszów, Poland; 7 Collegium Medicum, Institute of Nursing, University of Rzeszów, Rzeszów, Poland

**Keywords:** excessive workload, nurses, organizational factors, patient safety, work environment

## Abstract

**Background:**

Excessive workload among nurses constitutes a significant risk factor for adverse events, including medical errors, patient falls, and reduced quality of care. A growing body of evidence indicates that organizational factors play a key role in shaping the level of workload.

**Methods:**

A multicenter cross-sectional study was conducted between September 2024 and March 2025 among nurses working in public hospitals in Europe (*n* = 774). The study used an original questionnaire and the Subjective Workload Assessment Questionnaire (KONOP), which includes four dimensions: loss of control over work, perfectionist work style, general views on work, and perceived organizational oppressiveness. Statistical analyses included Student’s t-test, ANOVA variance analysis, and Pearson correlation coefficients.

**Results:**

Excessive workload among nurses remains closely associated with organizational factors that may affect the safety of both patients and staff. Interventions aimed at improving the organizational climate, the quality of management, and work organization may be of key importance for enhancing safety in healthcare systems.

**Conclusion:**

The excessive workload of nurses remains closely related to organizational factors that can affect patient and staff safety. Identifying and modifying working conditions, including work organization and organizational climate, is a key direction for actions improving safety in healthcare systems.

## 1 Introduction

Excessive workload among nurses is associated with an increased frequency of adverse events, such as medication administration errors, patient falls, and hospital-acquired infections, making it one of the key risk factors in healthcare systems. Numerous studies indicate that nurses’ working conditions, including excessive workload, have a direct impact on the quality of care, the risk of medical errors, and staff health (1–4). Modern nursing operates under conditions of increasing organizational demands, staff shortages, and pressure for efficiency, which promotes the accumulation of physical and psychosocial burdens. The *State of the World’s Nursing 2020* report emphasizes that investing in the work environment, leadership, and professional development of nurses is crucial for maintaining the capacity of health systems (1). At the same time, numerous reports indicate that overwork and chronic stress remain among the primary threats to the health of nursing staff (1–3).

Excessive workload not only reduces employee well-being but also increases the risk of adverse events, including medical errors, making it a significant problem in the area of health safety. According to the job demands–resources (JD-R) model, excessive demands combined with insufficient resources lead to impaired cognitive functioning, thereby increasing susceptibility to clinical errors and adverse events. Organizational factors, such as the work system, organizational climate, or level of control over work, can act as both a source of overload and an area for potential intervention to improve occupational safety (2). In an integrative review by Broetje et al., it was noted that key demands in nursing include workload, role conflict, and time pressure, while protective resources encompass supervisor support, organizational justice, and professional autonomy (2). This indicates that the work environment can act as both a risk factor and a protective factor.

The consequences of excessive workload extend beyond individual discomfort. The cohort study by Kelly et al. showed that increased levels of occupational burnout and staff turnover lead to team destabilization and may indirectly increase the risk of adverse events, thereby generating organizational costs and threatening continuity of care (3). Similar conclusions arise from studies conducted in Polish hospitals, where almost half of the nurses declared an intention to leave their place of employment, and environmental factors such as workload and emotional exhaustion were significantly associated with the intention to leave (4). These data confirm that overload is not merely an individual problem but a systemic one requiring intervention at the organizational level.

Particular attention is paid to working-time arrangements and shift systems. Long shifts, especially 12-h shifts, and limited control over work schedules may contribute to fatigue, reduced concentration, and increased emotional exhaustion, which in turn may increase the risk of medical errors and reduce the quality of care (5). Concurrently, studies indicate that the ability to participate in schedule planning and a sense of agency regarding working time can partially buffer the negative effects of workload (5). This means that not only the length of the shift but also the method of its organization is a potentially modifiable risk factor.

These factors constitute a key element of the so-called safety culture, which determines the frequency of adverse events in the clinical environment (6). Conversely, a positive work climate based on cooperation and transparency fosters well-being and engagement. In a longitudinal study by Larsman et al., it was shown that a climate of perceived organizational support is significantly associated with job satisfaction and lower burnout levels, and these relationships are prospective in nature (7). These results emphasize the importance of organizational support as a resource strengthening staff resilience.

Modern approaches to the health of medical personnel increasingly emphasize not only stress reduction but also the active enhancement of well-being and “flourishing” in the workplace. An integrative review by Bond et al. indicates that organizational factors such as leadership style, professional recognition, and development opportunities are key determinants of nurses’ well-being (8). From a clinical practice perspective, this implies a need to identify specific, modifiable elements of the work environment that can be targeted by managerial interventions.

Despite the growing number of studies on burnout and occupational stress, knowledge remains particularly limited regarding which components of excessive workload, such as loss of control over work or perceived organizational oppressiveness, are most important as risk factors for patient safety.

The present study is part of a larger project analyzing nurses’ workload in Central Europe; however, it focuses specifically on its significance in the context of patient and healthcare staff safety.

The aim of the study was to determine which components of excessive workload constitute the strongest risk indicators for the safety of patients and staff. Organizational factors were understood primarily as the country of employment, working-time system, and perception of the organizational environment, whereas the dimensions of excessive workload included loss of control over work (LCW), perfectionistic work style (PWS), general attitudes toward work (GAW), and perceived organizational oppressiveness (POO). The specific objectives were as follows:

- to assess differences in the levels of excessive workload components (LCW, PWS, GAW, POO) among nurses from Poland, Slovakia, and the Czech Republic in the context of their potential significance for patient and staff safety;
- to assess the impact of the work system on the level of excessive workload and to identify its association with risk factors in the work environment;
- to analyze interrelationships between the individual KONOP dimensions in order to determine their potential co-occurrence as complex risk factors;
- to identify organizational risk areas requiring intervention to improve patient safety and staff well-being.

## 2 Materials and methods

### 2.1 Research design

The presented results constitute a secondary data analysis derived from a larger multicenter research project conducted among nurses in three Central European countries. In contrast to previous analyses focusing on implications for human resource management, the present study focuses on identifying components of excessive workload as potential risk indicators for the safety of patients and healthcare staff.

A multicenter cross-sectional observational study was conducted using the diagnostic survey method. The project was designed to evaluate the relationship between selected organizational factors and the level of excessive workload among nurses. The use of a cross-sectional design enabled the simultaneous analysis of sociodemographic and organizational variables, as well as components of excessive workload within a large, diverse professional sample. The study was quantitative in nature and was based on the analysis of data obtained using a standardized psychometric tool and an original survey questionnaire. The study design was developed in accordance with the STROBE (Strengthening the Reporting of Observational Studies in Epidemiology) guidelines for observational studies (9).

### 2.2 Setting and period of the study

The study was conducted in healthcare entities operating within the healthcare systems of Central Europe. Data was collected between September 2024 and March 2025.

### 2.3 Participants

The study included female and male nurses actively practicing in inpatient care units. Inclusion criteria encompassed:

- Current employment as a nurse,
- Direct contact with patients or holding a managerial position within the nursing structure,
- Providing informed consent to participate in the study.

Only complete questionnaires from respondents were included in the analysis [*n* = 774]. The sample comprised individuals working in various working-time systems, including single-shift work, 8-h shift work, 12-h shift work, and mixed schedules, as well as in different positions, including staff nurse, coordinating nurse, and ward nurse.

### 2.4 Research tools

To assess the level of excessive workload, a standardized psychometric tool was used—the Subjective Workload Assessment Questionnaire (KONOP). The questionnaire consists of 65 items and includes four subscales: loss of control over work (16 items, *α* = 0.89), perfectionist work style (18 items, α = 0.83), general views on work (19 items, α = 0.90), and perceived organizational oppressiveness (12 items, α = 0.71). Respondents provided answers according to the tool’s instructions, and the results were analyzed as the sum of points for the individual subscales. Higher scores indicate a higher level of excessive workload in a given dimension.

For the purposes of the present study, language versions of the questionnaire were prepared for respondents from Slovakia and the Czech Republic. The adaptation process included translation from the original language into the target language and back-translation, followed by an assessment of semantic and substantive equivalence by experts in nursing and health sciences. The aim of this stage was to ensure the highest possible content equivalence between the language versions and to preserve the meaning of the individual items of the instrument.

Due to the cross-sectional design of the study and its multicenter organization, a full separate psychometric validation of the Slovak and Czech versions was not performed. Therefore, the Cronbach’s alpha coefficients reported in this paper refer to the original validation conducted for the Polish version. The questionnaire was used in the study with the consent of the author of the instrument (10, 11).

In the present study, the components of excessive workload (LCW, PWS, GAW, POO) were treated as potential risk indicators in the work environment, based on the assumption that higher values may be associated with a greater likelihood of adverse events and reduced occupational safety.

An original questionnaire was also utilized, covering sociodemographic and organizational variables, including age, gender, education, total work experience, work experience in the current place of employment, professional position, and working time system. These variables were treated as potential organizational factors, selected ones of which were included in the statistical analysis.

### 2.5 Data collection procedure

The questionnaires were distributed in paper format. Participation in the study was anonymous and voluntary. Before the study began, all participants received information about its purpose and provided informed, voluntary consent to participate. Respondents were informed that they could withdraw at any stage without providing a reason.

### 2.6 Statistical analysis

Calculations were performed using the IBM SPSS 29.0 statistical package. The adopted significance level was *p* = 0.05. During the analyses, one-way ANOVA, Student’s t-test for independent samples, and Pearson’s r correlation were utilized, following prior verification of the conditions of applicability for parametric tests. For the T-test and one-way ANOVA, when the homogeneity of variance assumption was violated, appropriate corrections and additional tests were applied. Additionally, an analysis was conducted to examine the relationships between organizational variables and the levels of individual components of excessive workload, which were treated as potential organizational determinants of risk.

### 2.7 Ethical procedure

The study was conducted in accordance with the principles of the Declaration of Helsinki. Approvals from the relevant bioethics committees were obtained: Poland (KBPANS/3/2024), Slovakia (UNV-40-29/224), and the Czech Republic (approval dated December 18th, 2024).

## 3 Results

### 3.1 Characteristics of the study group

The study included nurses from three Central European countries: Poland (*n* = 260), Slovakia (*n* = 256), and the Czech Republic (*n* = 258). Significant differences in average age were found between the groups. The lowest age was recorded among nurses from Poland (*M* = 40.00; *SD* = 11.11), while the highest was among nurses from Slovakia (*M* = 44.38; *SD* = 11.35). In the Czech group, the average age was 41.57 years (*SD* = 11.62) (Table 1).

**Table 1:** Sociodemographic characteristics of the study group.

Variable	Poland (*n* = 260)	Slovakia (*n* = 256)	Czech Republic (*n* = 258)	Statistical test
Age (years), M (SD)	40.00 (11.11)	44.38 (11.35)	41.57 (11.62)	*F*(2, 751) ≈ 10.5; *p* < 0.001
Work experience (years), M (SD)	14.34 (11.60)	19.33 (11.89)	17.93 (11.26)	F(2, 751) ≈ 14–16; *p* < 0.001
Tenure at current workplace (years), M (SD)	10.62 (10.13)	12.83 (10.21)	10.19 (8.12)	*F*(2, 751) ≈ 4–6; *p* < 0.01

One-way analysis of variance (ANOVA) showed statistically significant differences in age among the analyzed countries [*F*(2, 751) ≈ 10.5; *p* < 0.001]. The analysis of work experience indicated that the shortest average professional experience was found among nurses from Poland (*M* = 14.34 years; *SD* = 11.60), while the longest was in the Slovak group (*M* = 19.33 years; *SD* = 11.89). In the Czech Republic, average work experience was 17.93 years (*SD* = 11.26). ANOVA confirmed significant differences between countries regarding the length of work experience (*F*(2, 751) ≈ 14–16; *p* < 0.001).

Similar differentiation was observed regarding work experience in the current place of employment. The highest average tenure in one workplace was noted among nurses from Slovakia (*M* = 12.83; *SD* = 10.21). In Poland, the average was 10.62 years (*SD* = 10.13), while in the Czech Republic, it was 10.19 years (*SD* = 8.12). One-way analysis of variance showed significant differences between the studied countries (*F*(2, 751) ≈ 4–6; *p* < 0.01). The analysis of gender distribution using the chi-square test showed no statistically significant differences between the studied countries (*χ*^2^(2) ≈ 5.10; *p* > 0.05), indicating a comparable gender structure in the analyzed groups of nurses.

In all analyzed variables, a violation of the homogeneity of variance assumption was found (Levene’s test, *p* < 0.05), hence, in addition to standard ANOVA, robust tests (Welch and Brown-Forsythe) were applied. The obtained results were consistent across methods, confirming the stability of the analyses (Table 2).

**Table 2:** One-way analysis of variance (ANOVA) and descriptive statistics of the studied variables in the PL, SK, and CZ groups.

One-way analysis of variance (ONEWAY)		*N*	Mean	Standard deviation	Standard error	95% confidence interval for the mean		Minimum	Maximum	Levene’s Test	Significance	*F*	*p*	Welch	*p*	Brown-Forsythe	*p*	Effect size – Fixed effect Omega
						Lower bound	Upper bound											
Loss of control over work (16–80)	PL	260	42.96	10.52	0.65	41.68	44.25	19.00	69.00	11.28	0.000	4.76	0.009	4.27	0.015	4.77	0.009	0.010
	SK	256	40.77	7.85	0.49	39.80	41.74	18.00	69.00									
	CZ	258	40.73	9.66	0.60	39.55	41.92	21.00	67.00									
	Total	774	41.49	9.46	0.34	40.83	42.16	18.00	69.00									
Perfectionist work style (18–90)	PL	260	66.56	13.18	0.82	64.95	68.17	18.00	88.00	14.92	0.000	21.52	0.000	20.26	0.000	21.60	0.000	0.050
	SK	256	63.28	8.81	0.55	62.19	64.36	28.00	87.00									
	CZ	258	60.69	7.76	0.48	59.74	61.64	40.00	85.00									
	Total	774	63.52	10.47	0.38	62.78	64.26	18.00	88.00									
General views on work (19–95)	PL	260	47.65	11.13	0.69	46.30	49.01	20.00	85.00	12.70	0.000	6.28	0.002	8.04	0.000	6.29	0.002	0.013
	SK	256	48.95	9.14	0.57	47.82	50.07	20.00	87.00									
	CZ	258	46.02	7.47	0.46	45.11	46.94	27.00	70.00									
	Total	774	47.54	9.43	0.34	46.87	48.20	20.00	87.00									
Perceived oppressiveness of the organization (12–60)	PL	260	35.18	6.70	0.42	34.36	36.00	16.00	53.00	17.95	0.000	45.98	0.000	44.16	0.000	46.13	0.000	0.104
	SK	256	33.21	4.51	0.28	32.66	33.77	17.00	47.00									
	CZ	258	30.65	4.66	0.29	30.08	31.22	13.00	44.00									
	Total	774	**33.02**	5.69	0.20	32.61	33.42	13.00	53.00									

*N*, sample size; M, mean; SD, standard deviation; SE, standard error; 95% CI, 95% confidence interval for the mean; min, minimum; max, maximum. Homogeneity of variance was assessed with Levene’s test. In case of its violation, robust tests were applied (Welch and Brown-Forsythe). *F*, value of the test statistic; *p*, significance level. ω^2^ (omega squared) was interpreted as a measure of effect size (≈ 0.01, small effect, ≈ 0.06, medium effect, ≥ 0.14, large effect).

Differences between the groups were statistically significant across all dimensions of excessive workload (*p* < 0.05), but their practical significance was limited, with the exception of perceived organizational oppressiveness. Effect size analysis (ω^2^) indicated small effects in most dimensions and a moderate effect for organizational oppressiveness. Mean differences marked with asterisks indicate pairs of groups with statistically significant differences (*p* < 0.05). The most differences were observed regarding perceived organizational oppressiveness and perfectionist work style, while the fewest were for general views on work, where significant differences occurred only between the groups from the Czech Republic and Slovakia (p < 0.05). The greatest variation between countries concerned perceived organizational oppressiveness, highlighting the key role of organizational factors in shaping excessive workload.

Due to the violation of variance homogeneity, the Dunnett C *post hoc* test was used to analyze differences between pairs of groups, allowing the identification of statistically significantly different pairs (*p* < 0.05) (Table 3).

**Table 3:** Results of *post hoc* comparisons (mean differences) with Dunnett’s C test for the studied variables.

Dependent variable	Pair (I-J)	Mean difference (I-J)	Standard error	95% confidence interval (Lower)	95% Confidence interval (Upper)
Loss of control over work (16–80)	PL–SK	2.19*	0.82	0.27	4.12
	PL–CZ	2.23*	0.89	0.14	4.32
	SK–PL	−2.19*	0.82	−4.12	−0.27
	SK–CZ	0.04	0.78	−1.79	1.87
	CZ–PL	−2.23*	0.89	−4.32	−0.14
	CZ–SK	−0.04	0.78	−1.87	1.79
Perfectionist work style (18–90)	PL–SK	3.28*	0.99	0.96	5.61
	PL–CZ	5.87*	0.95	3.63	8.11
	SK–PL	−3.28*	0.99	−5.61	−0.96
	SK–CZ	2.58*	0.73	0.86	4.31
	CZ–PL	−5.87*	0.95	−8.11	−3.63
	CZ–SK	−2.58*	0.73	−4.31	−0.86
General views on work (19–95)	PL–SK	−1.29	0.90	−3.40	0.82
	PL–CZ	1.63	0.83	−0.33	3.59
	SK–PL	1.29	0.90	−0.82	3.40
	SK–CZ	2.92*	0.74	1.19	4.66
	CZ–PL	−1.63	0.83	−3.59	0.33
	CZ–SK	−2.92*	0.74	−4.66	−1.19
Perceived organizational oppressiveness (12–60)	PL–SK	1.97*	0.50	0.78	3.15
	PL–CZ	4.53*	0.51	3.33	5.72
	SK–PL	−1.97*	0.50	−3.15	−0.78
	SK–CZ	2.56*	0.40	1.61	3.52
	CZ–PL	−4.53*	0.51	−5.72	−3.33
	CZ–SK	−2.56*	0.40	−3.52	−1.61

*Correlation is significant at the 0.05 level (two-tailed).

An independent samples t-test revealed that the work system statistically significantly differentiated some domains of KONOP, but exclusively within the Slovak nursing staff group (*p* < 0.01). It was observed that higher means for perfectionist work style and general views on work occur in the case of a single-shift work system. A slightly more pronounced effect size estimated by Cohen’s d occurs for perfectionist work style (perfectionist work style–Cohen’s d = 0.713, general views on work–Cohen’s d = 0.464) (see Table 4).

**Table 4:** Pearson correlation coefficients between loss of control over work, perfectionist work style, general views on work, and perceived organizational oppressiveness in three countries (PL, SK, CZ).

**Group statistics**							Levene’s test for equality of variances		T-test for equality of means							Cohen’s d effect size
							F	Significance	t	df	p	Mean difference	Standard error of difference	95% confidence interval for the mean		
Group		7. Work system	N	**Mean**	Standard deviation	Standard error of the mean								Lower bound	Upper bound	
PL	Loss of control over work (16–80)	Single-shift	66	41.00	10.96	1.35	2.69	0.102	−1.76	258.00	0.079	−2.63	1.49	−5.57	0.31	−0.251
		Shift and mixed	194	43.63	10.31	0.74										
	Perfectionist work style (18–90)	Single-shift	66	64.94	15.25	1.88	5.57	0.019	−1.05	95.88	0.298	−2.17	2.08	−6.30	1.95	−0.165
		Shift and mixed	194	67.11	12.40	0.89										
	General views on work (19–95)	Single-shift	66	46.91	11.78	1.45	2.47	0.117	−0.63	258.00	0.530	−1.00	1.59	−4.12	2.13	−0.090
		Shift and mixed	194	47.91	10.91	0.78										
	Perceived oppressiveness of the organization (12–60)	Single-shift	66	34.58	7.52	0.93	1.45	0.229	−0.84	258.00	0.400	−0.81	0.96	−2.69	1.08	−0.120
		Shift and mixed	194	35.38	6.41	0.46										
SK	Loss of control over work (16–80)	Single-shift	72	42.04	8.49	1.00	0.88	0.349	1.63	254.00	0.105	1.77	1.09	−0.37	3.91	0.226
		Shift and mixed	184	40.27	7.55	0.56										
	Perfectionist work style (18–90)	Single-shift	72	67.58	8.95	1.05	0.02	0.895	5.13	254.00	0.000	5.99	1.17	3.69	8.29	0.713
		Shift and mixed	184	61.59	8.19	0.60										
	General views on work (19–95)	Single-shift	72	51.93	8.20	0.97	1.28	0.259	3.33	254.00	0.001	4.15	1.25	1.70	6.61	0.464
		Shift and mixed	184	47.78	9.24	0.68										
	Perceived oppressiveness of the organization (12–60)	Single-shift	72	32.56	4.61	0.54	0.12	0.732	−1.46	254.00	0.146	−0.91	0.63	−2.14	0.32	−0.203
		Shift and mixed	184	33.47	4.45	0.33										
CZ	Loss of control over work (16–80)	Single-shift	63	39.11	10.27	1.29	0.36	0.547	−1.54	256.00	0.126	−2.15	1.40	−4.89	0.60	−0.223
		Shift and mixed	195	41.26	9.42	0.67										
	Perfectionist work style (18–90)	Single-shift	63	60.16	7.77	0.98	0.19	0.660	−0.63	256.00	0.530	−0.71	1.13	−2.92	1.51	−0.091
		Shift and mixed	195	60.87	7.76	0.56										
	General views on work (19–95)	Single-shift	63	46.65	7.19	0.91	0.08	0.772	0.77	256.00	0.444	0.83	1.08	−1.30	2.96	0.111
		Shift and mixed	195	45.82	7.56	0.54										
	Perceived oppressiveness of the organization (12–60)	Single-shift	63	30.16	5.38	0.68	3.99	0.047	−0.86	90.41	0.390	−0.65	0.75	−2.13	0.84	−0.139
		Shift and mixed	195	30.81	4.41	0.32										

*N*, sample size; *M*, mean; SD, standard deviation; SE, standard error of the mean. Levene’s test is used to assess the homogeneity of variance; in case of its violation, the results of the *t*-test with a correction for unequal variances (Welch) were interpreted. *t*, value of the Student’s *t*-test statistic; df, degrees of freedom; *p*, level of statistical significance; mean difference, difference between the compared groups; SE of the difference, standard error of the difference; 95% CI, 95% confidence interval for the difference of means. Cohen’s d, measure of effect size (≈ 0.20, small effect, ≈ 0.50, medium, ≥ 0.80, large effect).

In the groups of respondents from Poland, Slovakia, and the Czech Republic, numerous statistically significant positive correlations (*p* < 0.05) were observed between individual domains of the KONOP questionnaire, and most of them have at least a moderate strength of Pearson’s r association. The strongest correlation coefficients in Poland indicate that higher scores for general views on work and perceived organizational oppressiveness are associated with higher scores for the loss of control over work. As scores for perceived organizational oppressiveness increase, scores for general views on work also increase. Additionally, higher levels of loss of control over work and general views on work are linked to higher scores of perceived organizational oppressiveness.

For respondents from the Czech Republic, it is clearly visible that higher scores for general views on work correspond to higher scores for loss of control over work and perfectionist work style. Furthermore, with an increase in scores for the loss of control over work and perfectionist work style, scores for general views on work also rise. In the group of respondents from Slovakia, correlation coefficient values are characterized by, at most, moderate association strengths. Effects were moderate to large, especially in the area of perfectionist work style (see Table 5).

**Table 5:** Pearson correlation coefficients between loss of control over work, perfectionist work style, general views on work, and perceived organizational oppressiveness in three countries (PL, SK, CZ).

Group	Dimension	Loss of control over work (16–80)	Perfectionist work style (18–90)	General views on work (19–95)	Perceived organizational oppressiveness (12–60)
PL (*n* = 260)	Loss of control over work	1	0.443**	0.650**	0.539**
	Perfectionist work style	0.443**	1	0.487**	0.306**
	General views on work	0.650**	0.487**	1	0.522**
	Perceived organizational oppressiveness	0.539**	0.306**	0.522**	1
SK (*n* = 256)	Loss of control over work	1	0.286**	0.476**	0.221**
	Perfectionist work style	0.286**	1	0.396**	−0.009
	General views on work	0.476**	0.396**	1	0.297**
	Perceived organizational oppressiveness	0.221**	−0.009	0.297**	1
CZ (*n* = 258)	Loss of control over work	1	0.470**	0.582**	0.435**
	Perfectionist work style	0.470**	1	0.548**	0.124*
	General views on work	0.582**	0.548**	1	0.466**
	Perceived organizational oppressiveness	0.435**	0.124*	0.466**	1

*Correlation is significant at the 0.05 level (two-tailed).

**Correlation is significant at the 0.01 level (two-tailed).

## 4 Discussion

The most important finding of the present study is the identification of perceived organizational oppressiveness as the strongest component of excessive workload with potential relevance to patient and staff safety. Although most effects were statistically significant, their magnitude was small, suggesting limited practical significance, with the exception of perceived organizational oppressiveness.

The greatest between-country variation was observed precisely for perceived organizational oppressiveness, suggesting that the perception of the work environment—including the level of support, organizational justice, quality of communication, and institutional pressure—may constitute one of the main mechanisms associated with excessive workload. These findings are consistent with reports indicating that organizational culture and work climate are among the strongest predictors of occupational stress and overload among nurses (6, 12). At the same time, more recent studies show that the work environment is associated not only with stress levels but also with patient safety culture and the safety of nursing activities (13–15). In this context, our findings suggest that perceived organizational oppressiveness may be treated as a practical indicator of organizational risk, understood as an increased likelihood of staff overload, deterioration of occupational well-being, and reduced safety of care.

An important element of the findings is also the relationship between the individual dimensions of excessive workload. The strong positive correlations between perceived organizational oppressiveness and loss of control over work, as well as general attitudes toward work, may indicate the co-occurrence and mutual reinforcement of these phenomena. This suggests that work overload does not occur in isolation but has a cumulative character, which may increase the risk of clinical errors through the simultaneous influence of multiple factors. In terms of the job demands–resources (JD-R) model, excessive demands combined with insufficient resources may lead to exhaustion, impaired cognitive functioning, and reduced adaptive capacity of the employee (2). This relationship is supported by studies showing that work-related stress may reduce safety practices and weaken patient safety culture (16, 17). Similarly, Wang et al. showed that the work environment and occupational stress among newly graduated nurses are associated with their attitudes toward patient safety (17).

Our findings should also be interpreted in light of studies indicating mediating mechanisms between working conditions and clinical and organizational outcomes. Niri et al. (18) demonstrated that nurses’ fatigue reduces their work effectiveness, which in turn mediates the relationship between fatigue and patient safety culture. In turn, Furlan and Guirardello (19) indicated that job satisfaction partially mediates the relationship between the work environment and occupational burnout. This suggests that work organization may exert its effects not only directly but also through more complex psychological and occupational pathways. In this sense, the relationships observed in our study between organizational oppressiveness, loss of control over work, and perfectionistic work style may reflect a complex, multistage mechanism of overload.

An interesting finding of the study is the differentiated relationship between the work system and workload level, which proved significant only in the Slovak group. In this group, nurses working in a single-shift system obtained higher scores for perfectionistic work style and general attitudes toward work. This finding suggests that the significance of the work system is not universal but depends on the broader organizational and cultural context. This is consistent with more recent reports indicating that not only the shift itself or its length, but also the degree of control over the schedule, workload related to patient safety tasks, and flexibility of work organization may influence subjectively perceived workload (5, 20, 21). Hong further demonstrated that patient safety-related activities themselves may constitute an additional source of workload among nurses working shifts, which should be considered when planning staffing and work organization (22).

It is worth emphasizing that the highest levels of perfectionistic work style and perceived organizational oppressiveness were recorded in Poland, whereas the lowest were recorded in the Czech Republic. This may indicate systemic differences in work organization, management culture, and leadership practices. The literature increasingly emphasizes that professional autonomy and the quality of the work environment are recognized as important protective factors for patient safety (19, 20). Yuk and Yu demonstrated that both professional autonomy and the work environment are associated with nurses’ patient safety activities, which corresponds well with our finding indicating the importance of organizational oppressiveness as the opposite of a supportive environment (23).

The consequences of excessive workload extend beyond individual discomfort and may affect the functioning of entire healthcare organizations. Occupational burnout and staff overload are associated with deterioration in the quality of care and an increased risk of adverse events, and may also indirectly destabilize continuity of care through increased staff turnover (3, 4, 24, 25).

Our findings are also consistent with studies indicating that nurses’ work organization is associated with physical, mental, and emotional workload. Ferramosca et al. (26) emphasize that workload is simultaneously influenced by patient-related, nurse-related, and workflow-related factors. This is important in the context of our study, as it suggests that the observed differences between countries may result not only from the overall organizational culture but also from everyday patterns of work organization on hospital wards.

Contemporary studies further show that a poor nursing work environment is associated with poorer mental health, sleep disturbances, and reduced well-being (17–19). Edwin et al. indicate that improving the work environment and strengthening health-promoting behaviors may reduce psychological stress among staff (20). At the same time, the review by Widayana et al. highlights the importance of work flexibility, social support, and measures supporting work–life balance (21). Incorporating these elements into staff management strategies may be important not only for employee well-being but also for the quality of care and workforce stability.

Reports from intensive care and critical care settings also deserve attention. Erbay Dalli showed that teamwork climate, perceptions of management, job satisfaction, and the ability to cope with stress are significantly associated with work performance and attitudes toward patient safety (15). In turn, Al Ma’mari et al. showed that fatigue, burnout, work environment, and workload remain significantly associated with overall patient safety (27). These findings strengthen the interpretation of our data, according to which the organizational determinants of nurses’ work are closely intertwined with clinical safety.

Finally, the review by Pérez-Francisco et al. indicates that high workload among nurses is associated with deterioration of their health, higher levels of burnout, lower quality of care, and poorer patient safety (24). Combined with our findings, this suggests that excessive workload should be treated as a multidimensional phenomenon: psychological, organizational, and related to public safety at the same time.

The present study extends previous approaches by providing a multidimensional analysis of work overload and identifying organizational oppressiveness as a central mechanism of this phenomenon. The findings confirm that excessive workload is not solely an individual problem but a systemic one. Work organization, institutional culture, professional autonomy, quality of support, and staff management practices may constitute key factors associated with both employee well-being and patient safety. In light of contemporary research, effective interventions should include not only the reduction of demands but also the strengthening of organizational resources, such as supervisor support, transparency of decision-making, participation in work planning, and improvement of the practical work environment (8, 12, 13, 15, 16, 22, 23).

The findings indicate that excessive workload among nurses should be interpreted as a systemic phenomenon, strongly rooted in work organization and the perception of the professional environment. Perceived organizational oppressiveness is of particular importance, as it may constitute a key risk indicator for patient and staff safety. In practice, this implies the need to shift interventions from the individual level to the organizational level, including improvement of the work climate, support, and transparency of management.

## 5 Limitations of the study

The study has several limitations that must be considered when interpreting the obtained results. First, the cross-sectional study design prevents the determination of causal relationships between the analyzed variables. The results only point to the co-occurrence of specific phenomena. Second, data were collected via self-report methods, which may involve response bias and the influence of subjective factors, such as the respondents’ current emotional state. This could lead to an over- or under-estimation of workload and organizational environment perceptions. Another limitation is the sampling method, which was purposive and involved selected hospitals in three Central European countries. Despite the relatively large sample size, the results cannot be directly generalized to the entire nursing population in the studied nations. It should also be noted that the study focused on selected organizational factors and did not encompass all potential variables that might impact excessive workload levels, such as detailed ward working conditions, management style, or patient load. Furthermore, although various work systems were included, their classification was simplified and lacked detailed parameters like shift length or the number of night shifts, which could significantly affect overload. In light of the above limitations, the findings should be interpreted with caution, particularly with regard to causal inference and the possibility of fully generalizing the results to the population of nurses in other countries and healthcare systems.

## 6 Implications for practice

The results indicate that the excessive workload of nurses has substantial organizational determinants, which can be the target of interventions at the level of healthcare system management and individual medical facilities. Of particular importance is perceived organizational oppressiveness, which proved to be the strongest differentiating factor among the countries and may serve as a key area for remedial action. Practically, this entails the necessity of implementing strategies aimed at improving the organizational climate, including enhancing supervisor support, increasing decision-making transparency, and building an organizational culture rooted in trust and cooperation. Such interventions can help mitigate the feeling of organizational pressure and reduce the risk of work overload.

Working time organization is also a vital action area. Although the impact of the work system varied by country, the findings suggest that schedule organization methods and employees’ control over their working time may shape work attitudes and overload levels. Implementing flexible solutions that allow greater employee participation in work planning can be an essential preventive element.

The results also highlight the need to monitor and curb excessive perfectionism as a professional functioning style, which—while often deemed desirable—can foster work overload and impair staff well-being. In this context, promoting realistic work standards and supporting a balance between employee demands and resources is crucial. From a systemic perspective, the findings underscore the necessity of treating excessive workload not merely as an individual issue but as an organizational phenomenon with direct consequences for patient safety. Implementing comprehensive programs to improve the work environment can help raise care quality, limit staff turnover, and enhance the overall efficiency of healthcare systems.

## 7 Conclusion

The excessive workload of nurses is a significant organizational problem affecting patient safety and staff well-being. Organizational factors, especially perceived oppressiveness of the work environment, play a pivotal role. The disparities between countries primarily concern these aspects, emphasizing the importance of organizational culture and management style. The impact of the work system was context-dependent, proving significant only within the Slovak group. Perceived organizational oppressiveness may be treated as a practical risk indicator in the assessment of workplace safety. The interconnections between workload dimensions signal its complex nature, particularly in the relationship between organizational oppressiveness and the loss of control over work. The findings suggest a need for initiatives that improve working conditions, increase support and autonomy, and optimize working time organization.

## Data Availability

The raw data supporting the conclusions of this article will be made available by the authors, without undue reservation.
