# Supplementary material for: Excessive workload as a risk factor for patient and medical staff safety: a multicenter cross-sectional study in Central Europe
Source: Front Public Health. 2026 May 28;14:1851838. doi: 10.3389/fpubh.2026.1851838 (PMC13253311; doi:10.3389/fpubh.2026.1851838)
Supplement: Supplementary file 1 [file Supplementary_file_1.docx]

**STROBE CHECKLIST**

**Title of the paper:** Workload as a Risk Factor for Patient and Medical Staff Safety: A Multicenter Cross-Sectional Study in Central Europe

**Type of study:** Cross-sectional study

| **No.** | **STROBE item** | **Met** | **Location in the article** |
| --- | --- | --- | --- |
| 1 | Title/abstract – indication of the study design | ✔ | Title, abstract |
| 2 | Title/abstract – balanced description of methods and results | ✔ | Abstract |
| 3 | Introduction – rationale for the study | ✔ | Introduction |
| 4 | Study objectives and hypotheses | ✔ | End of introduction |
| 5 | Study design | ✔ | Methods 2.1 |
| 6 | Setting, location, recruitment period | ✔ | Methods 2.2 |
| 7 | Participants – eligibility criteria and sample selection | ✔ | Methods 2.3 |
| 8 | Variables – definition of dependent, independent, and confounding variables | ✔ | Methods 2.3–2.4 |
| 9 | Data sources and measurement methods | ✔ | Methods 2.4 |
| 10 | Bias – measures to address systematic errors | ✔ | Methods 2.4, Limitations |
| 11 | Study size | ✔ | Methods 2.3 |
| 12 | Quantitative variables | ✔ | Methods 2.6 |
| 13 | Statistical methods | ✔ | Methods 2.6 |
| 14 | Subgroup and interaction analyses | ✔ | Methods 2.6 |
| 15 | Missing data | ✔ | Methods 2.3 |
| 16 | Analyses accounting for the sampling strategy / sensitivity analyses | ✔ | Methods 2.6 |
| 17 | Participants – numbers at each stage of the study | ✔ | Results 3.1 |
| 18 | Reasons for exclusion or non-participation | ✔ | Methods 2.3 |
| 19 | Descriptive data on participants | ✔ | Results 3.1, Table 1 |
| 20 | Main results | ✔ | Results, Tables 2–4 |
| 21 | Additional analyses | ✔ | Results 3 |
| 22 | Summary of results | ✔ | Discussion |
| 23 | Study limitations | ✔ | Study limitations |
| 24 | Interpretation of results | ✔ | Discussion |
| 25 | Generalizability of results | ✔ | Limitations, Practical implications |
| 26 | Funding | ✔ | Separate section / if applicable |

**Based on:** von Elm E, Altman DG, Egger M et al. The Strengthening the Reporting of Observational Studies in Epidemiology (STROBE) Statement: Guidelines for Reporting Observational Studies.
